# An integrated knowledge translation (iKT) approach to advancing community-based depression care in Vietnam: lessons from an ongoing research-policy collaboration

**DOI:** 10.1186/s12913-023-10518-3

**Published:** 2024-01-27

**Authors:** Jill K. Murphy, Leena W. Chau, Vu Cong Nguyen, Harry Minas, Duong Viet Anh, John O’Neil

**Affiliations:** 1 Department of Psychiatry, Faculty of Medicine, The University of British Columbia, Vancouver, Canada; 2 Faculty of Health Sciences, Simon Fraser University, Vancouver, Canada; 3 Institute of Population, Health and Development, Hanoi, Vietnam; 4 Global and Cultural Mental Health Unit, Centre for Mental Health, Melbourne School of Population and Global Health, University of Melbourne, Melbourne, Australia

**Keywords:** Global mental health, Integrated knowledge translation, Policy engagement, Stakeholder engagement, Collaboration, Case study

## Abstract

**Background:**

Evidence-based mental health policies are key to supporting the expansion of community-based mental health care and are increasingly being developed in low and middle-income countries (LMICs). Despite this, research on the process of mental health policy development in LMICs is limited. Engagement between researchers and policy makers via an integrated Knowledge Translation (iKT) approach can help to facilitate the process of evidence-based policy making. This paper provides a descriptive case study of a decade-long policy and research collaboration between partners in Vietnam, Canada and Australia to advance mental health policy for community-based depression care in Vietnam.

**Methods:**

This descriptive case study draws on qualitative data including team meeting minutes, a focus group discussion with research team leaders, and key informant interviews with two Vietnamese policy makers. Our analysis draws on Murphy et al.’s (2021) findings and recommendations related to stakeholder engagement in global mental health research.

**Results:**

Consistent with Murphy et al.’s findings, facilitating factors across three thematic categories were identified. Related to ‘the importance of understanding context’, engagement between researchers and policy partners from the formative research stage provided a foundation for engagement that aligned with local priorities. The COVID-19 pandemic acted as a catalyst to further advance the prioritization of mental heath by the Government of Vietnam. ‘The nature of engagement’ is also important, with findings demonstrating that long-term policy engagement was facilitated by continuous funding mechanisms that have enabled trust-building and allowed the research team to respond to local priorities over time. ‘Communication and dissemination’ are also crucial, with the research team supporting mental health awareness-raising among policy makers and the community, including via capacity building initiatives.

**Conclusions:**

This case study identifies factors influencing policy engagement for mental health system strengthening in an LMIC setting. Sustained engagement with policy leaders helps to ensure alignment with local priorities, thus facilitating uptake and scale-up. Funding agencies can play a crucial role in supporting mental health system development through longer term funding mechanisms. Increased research related to the policy engagement process in global mental health will further support policy development and improvement in mental health care in LMICs.

**Supplementary Information:**

The online version contains supplementary material available at 10.1186/s12913-023-10518-3.

## Background

### Introduction

The shift from institutional towards community-based models of mental health care began in many high income countries (HICs) in the mid twentieth century and has been recommended by the World Health Organization (WHO) for over two decades [1, 2] as an approach to improving access to and quality of care and promoting the human rights of people with mental health conditions in lower resourced settings [3, 4]. Models of care including the integration of mental health services into primary care and task-sharing, whereby mental health care is provided by non-specialist providers, are recommended to support increased community-based capacity for mental health support, particularly in areas with low availability of mental health human resources [5–7]. Despite this recommendation, progress towards the implementation and scale-up of community-based mental health care in much of the world has been slow [4].

Evidence-based mental health policies have been identified as a key factor in the successful development and implementation of appropriate community-based care programs [4, 8]. In the last two decades, low-and-middle income countries (LMICs) are increasingly developing mental health policies, legislation and plans, often with the technical support of partners in HICs [9]. The inclusion of mental health considerations in the United Nations’ Sustainable Development Goals, among other initiatives by multilateral organizations such as WHO [1, 10], encouraged the inclusion of mental health in national health, social and economic policies in many countries [5]. Despite a rapid increase in mental health policy development, research from LMICs on the policy development process remains relatively limited [9]. Further investigation of this often complex process is necessary to inform similar initiatives to support the development of community-based models of care.

Engagement with policy makers in mental health research is recognized as an important approach that facilitates the alignment of research with local priorities and promotes the uptake and scale-up of research results [11, 12]. Integrated Knowledge Translation (iKT) is a collaborative approach to engagement with ‘knowledge users’, including policy makers. In an iKT approach, knowledge users are engaged as research collaborators, often from the inception of the research, helping to set priorities [13]. Though iKT can lead to the improved translation of research into policy [13], the approach can also be challenging. In a qualitative study undertaken by Murphy et al. [11] identifying barriers to and drivers of stakeholder engagement [11] in global mental health (GMH) implementation projects, participants described several challenges related to policy engagement. Challenges include lack of training, capacity and resources among researchers to support policy engagement and low levels of knowledge and understanding about mental health by policy makers leading to low prioritization of mental health. Murphy et al. [11] identified three broad findings and made related recommendations to facilitate effective engagement. Findings relevant to policy makers are adapted from Murphy et al. [11] and presented in Table 1.

**Table 1:** Findings and recommendations to facilitate policy maker engagement in GMH research [11]

**Finding**	**Recommendations**
The importance of understanding context	Invest in high quality formative research
	Explore crises as a catalyst for positive change
The nature of engagement	Invest adequate time and funding to support engagement and trust-building
	Create opportunities for meaningful and active engagement by end users, providers and policy makers
	Leverage existing resources and relationships
Communication and dissemination	Invest in informed mental health awareness raising and communication strategies
	Create mechanisms to support engagement from program inception
	Invest in capacity development opportunities to support knowledge translation and communications activities by researchers Invest in activities that promote mental health capacity building of policy makers

This paper provides a descriptive case study of a decade-long policy and research collaboration between partners in Vietnam, Canada and Australia to advance mental health policy for community-based depression care in Vietnam. Drawing on the concepts of iKT and the above recommendations for policy engagement, we explore factors that have contributed to policy development as well as challenges faced in the ongoing mental health policy and practice context, including the COVID-19 pandemic.

### Policy engagement and depression research in Vietnam

**Fig. 1:**
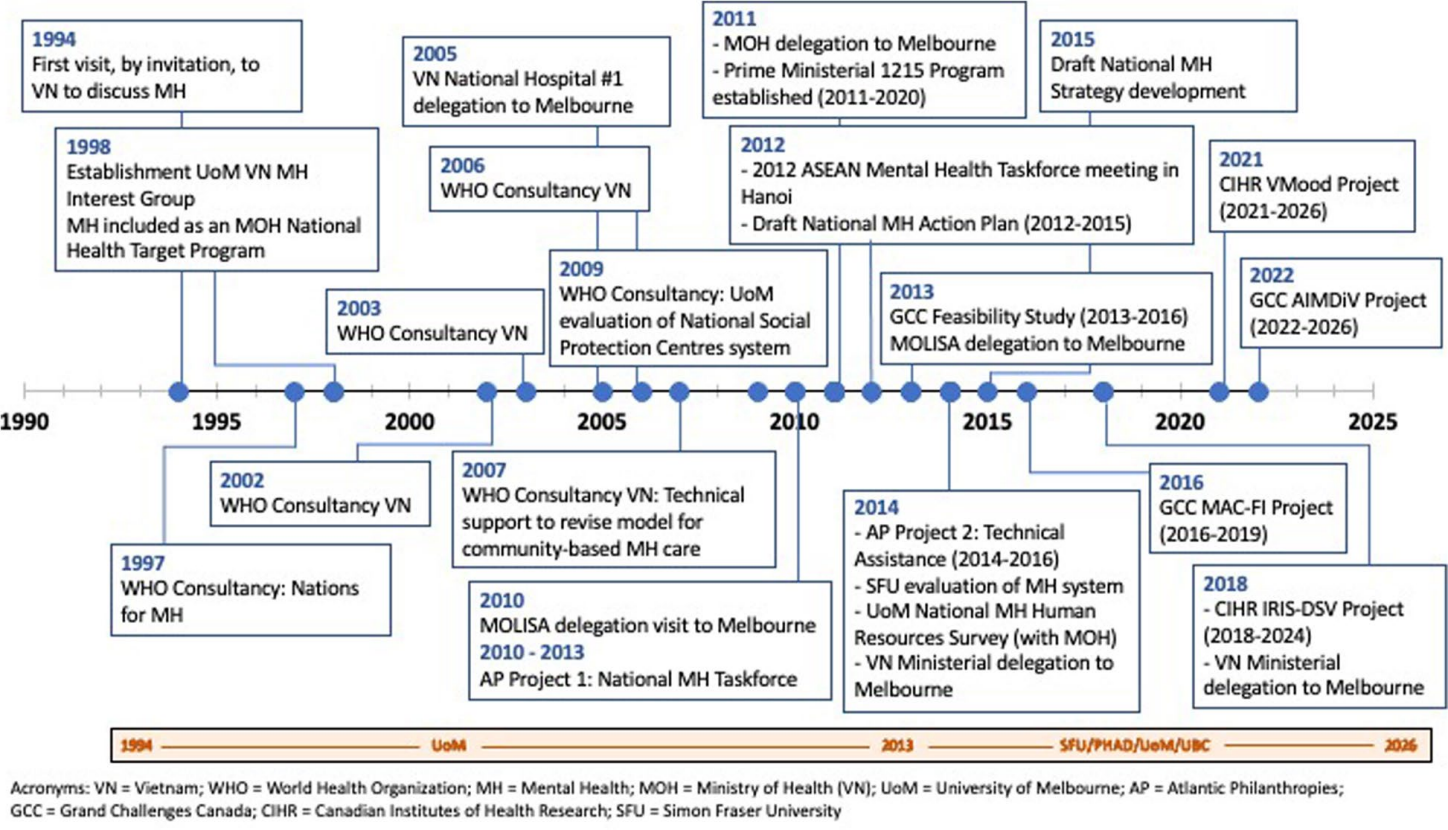
Key milestones, activities and studies

This case study describes an ongoing research and policy collaboration between partners in Canada (Simon Fraser University-SFU; University of British Columbia-UBC), Australia (University of Melbourne-UoM), and Vietnam (Institute of Population, Health and Development-PHAD; Ministry of Labour, Invalids and Social Affairs-MOLISA, and the Ministry of Health-MoH) and its contribution to mental health system strengthening in Vietnam. Specifically, this partnership, which commenced in 2013, has focused on developing community-based care for common mental disorders including depression and anxiety. More detail about the broader mental health context in Vietnam can be found elsewhere [14]. Key milestones, activities and studies that have involved at least one member from the university partners across almost three decades of policy engagement in Vietnam are described in Fig. 1.

Vietnam has a population of approximately 98 million people and is made up of 63 provinces. Vietnam’s mental health system consists primarily of an institutional approach largely focused on treating severe mental health conditions, with care delivered at tertiary hospitals and very little or no care available in the community. Like in many LMICs, Vietnam has very limited mental health human resources, with fewer than two psychiatrists per 100,000 population [15]. There are 36 psychiatric hospitals and 25 psychiatry departments in provincial general hospitals under the leadership of MoH [16]. There are also 24 long term care centres for people with severe mental health conditions in the Provincial Social Protection Centres, under the jurisdiction of MOLISA. Primary care services currently play a limited role in the mental health system, consisting of detection and referral to tertiary care for formal diagnosis, prescriptions and inpatient care [17, 18]. While Vietnam does have a Community Mental Health Program (CMHP), coverage is limited and it primarily consists of referrals and dispensing medication for severe mental illness and epilepsy following a diagnosis and prescription from a tertiary facility [19, 20]. Mental health care for common mental disorders including mild to moderate depression and anxiety is severely limited in the country.

At a policy level the Vietnamese government began dedicating attention to the enhancement of community-based mental health care, including for common mental disorders, in the late 1990s. The first shift towards a community-based approach was initiated in 1998 when the MoH first included mental health as a National Target Program, with the CMHP beginning in 2000 [14, 18, 20]. The evolution of mental health policy in Vietnam and the roles played by MoH, MOLISA and several international partners is described in detail elsewhere [14, 18].

The current policy and research partnership builds on extensive mental health system development work in Vietnam led by the UoM beginning in 1994 [21–23] (see Fig. 1). This work has included WHO consultancies on mental health system development, leadership capacity building initiatives for senior leaders, conferences and meetings in Vietnam, several visits by senior Vietnamese delegates from ministries of health, social affairs and finance to Melbourne to learn about mental health policy and services, and several research and policy projects [18]. Several components of this mental health system development work were funded over more than 10 years through multiple grants from the Australian Agency for International Development (AusAID) and, between 2010 and 2016, by Atlantic Philanthropies (AP). As an example of the policy impact of these initiatives, a WHO-commissioned evaluation of social protection centres [24] was the basis for the development of the 1215 National Program on Community Based Social Support and Rehabilitation for People with Mental Disorders (2010–2020), which emphasized a shift from long-term institutionalization of people with severe mental illness towards programs to support their integration into the community. The extensive contributions of UoM and partners to the development of mental health policy in Vietnam is described elsewhere [14, 18].

In 2013, SFU established a partnership with PHAD and UoM to conduct a Grand Challenges Canada (GCC)-funded pilot study to adapt a Canadian-developed Supported-Self Management (SSM) intervention for depression for use in Vietnam and to assess the feasibility of conducting a randomized controlled trial of its delivery in community-based settings [25]. PHAD is a leading non-governmental public health research institute in Vietnam with a long history of collaboration with MOLISA, MoH and other ministries on initiatives that have informed the development and implementation of evidence-based health policy in the country. In 2013, PHAD began providing technical assistance to MOLISA and MoH to support Vietnam’s National Mental Health Program. During the feasibility study, PHAD and SFU engaged with representatives of MOLISA’s Department of Social Protection and with MoH. This engagement coincided with MOLISA’s ongoing priority of expanding mental health care with the support of social workers and lay social workers (called ‘social collaborators’), leading to MOLISA expanding the pilot study intervention from two districts of Hanoi to two additional provinces and further strengthening the collaboration with MOLISA.

In 2016, following promising results from the feasibility study [25], SFU, PHAD and UoM received transition-to-scale funding from GCC to conduct a randomized controlled trial (RCT) of the SSM intervention. MOLISA contributed CAD $549,000 in matched funding to this study, demonstrating their commitment to enhancing community-based care for depression. This study took place from 2016-2019 and demonstrated the effectiveness of the intervention [26] and the potential of engaging lay social workers in the delivery of community-based mental health care via a task-sharing model [27].

In 2018, the team was successful in obtaining five years of funding from the Canadian Institutes of Health Research (CIHR) to examine ‘real world’ implementation factors influencing the scale-up of the SSM intervention in Vietnam, including the mental health policy context. At the same time, MOLISA was preparing to evaluate the aforementioned 1215 National Program and to plan for the development of a subsequent policy for 2021-2030. In 2019, UoM, PHAD and SFU were engaged to conduct a comprehensive evaluation of the 1215 National Program and to support the development of a proposal for the program post-2020. The evaluation of the 1215 program consisted of an extensive review of national and provincial reports and documents and of key informant interviews with representatives of MOLISA and MoH and was finalized in early 2020. Collaboration on the proposal for the post-2020 program was slightly delayed due to the COVID-19 pandemic, but in late 2020 Decision 1929 Approval for Social Assistance and Community Based Rehabilitation for the Mentally Ill, Autistic Children and People with Mental Disorders (2021- 2030) was signed by the Prime Minister. The budget commitment for the first five years of development of a new model of community-based care for people with mental health conditions, autism and disabilities is approximately 500 billion VND (USD22 million) with an additional USD108 million committed for replication (scale-up) in the 2025-2030 period.

Following the 2016-2019 SSM RCT which demonstrated the effectiveness of the in-person intervention, MOLISA expressed interest in exploring opportunities to deliver the intervention online as a more cost-effective and feasible approach to scaling up the program. The potential of using digital technologies to implement the intervention was further emphasized during the COVID-19 pandemic, when restrictions to face-to-face interactions led to a rapid shift to the use of tele- and e-health approaches worldwide. Our team received funding from CIHR (2021) and GCC (2022) to: adapt the in-person SSM intervention for delivery via a smartphone app (VMood); test its effectiveness compared with the in-person version of the intervention via an RCT; conduct a cost effectiveness analysis of the intervention; and support MOLISA in strengthening its mental health information system, which, like in many LMICs, is not well-developed. The study protocol, which describes each study objective in detail, is published elsewhere [28]. In alignment with Decision 1929 and the related budget commitment for enhancing community-based mental health care, MOLISA is again partnering in this study, providing matched funding with GCC, and has committed to scaling up the app-based intervention should the study demonstrate its effectiveness.

## Methods

### Aims and framework

This paper presents a descriptive qualitative case study that highlights key factors that have facilitated sustained research and policy engagement in Vietnam over the past decade that has aimed to improve community-based depression care, as described above and in Fig. 1. Qualitative analysis draws on the findings and recommendations to facilitate stakeholder engagement in GMH developed by Murphy et al. [11], as described in Table 1. Results from this case study are mapped onto the three key themes and recommendations identified in the paper.

### Data collection

The first of five studies began in 2013 (to 2016), with the most recent study funded in 2022 (to 2026). Three of the studies are ongoing at the time of writing this paper. See Table 2 for a summary of the studies. This descriptive case study draws on internal personal communications, a focus group with study team members and interviews with policy partners in Vietnam. The first source of data is verbal conversations between study team members, captured via emails and team meeting minutes, on their experiences with the research-policy collaboration across the five studies. The second is a focus group conducted in English by co-lead author LC with key research team members (HM, NVC, JON) who have been involved since development of the first study in 2013. During this focus group, participants were asked to describe the long history of policy engagement in Vietnam including how the relationship with MOLISA was formed, key barriers and drivers that supported the engagement process, along with the results from that process. Field notes were gathered from the focus group to capture the discussion.

**Table 2:** Summary of the studies

**Name**	**Objective(s)**	**Funder**	**Dates**
Feasibility study in preparation for randomized control trial	To test the feasibility of: a) Introducing a supported self-management (SSM) intervention for depression in Vietnam; b) Conducting an RCT to test the effectiveness of the SSM intervention.(1)	GCC	December 2013 – December 2016 (3 years)
**MAC-FI**: Mental Health in Adults and Children – Frugal Innovations	To test the effectiveness of the SSM intervention at reducing symptoms of depression among adults in community-based and primary care settings in Vietnam.(2)	GCC	Phase 1: March – December 2016 Phase 2: March 2017 – March 2019
**IRIS-DSV**: Implementation Research to Improve Scale-up of Depression Services in Vietnam	To identify factors influencing the effectiveness of delivery of SSM by lay providers in Vietnam and identify areas relevant for scale-up.(3)	CIHR	April 2018 – March 2023
**VMood**: Implementing an mHealth App to Manage Depression in Vietnam	a) To establish key elements of implementation fidelity when adapting SSM to a digital, app-based format (VMood); b) To explore the usability and acceptability of VMood for implementation in the Vietnamese context	CIHR	October 2021 – March 2025
**AIMDiV**: Accessing Innovative Mental Health Services for Depression in Vietnam	To test the effectiveness and cost-effectiveness of a digital, app-based intervention for depression (VMood), adapted from an in-person SSM intervention.(4)	GCC	March 2022 – March 2026

We also conducted two semi-structured interviews with our long-time collaborators from MOLISA, a Vice Minister and the Acting Director of the Social Protection Department, to examine the Government of Vietnam’s commitments to mental health that supported policy engagement and to further explore the challenges and enablers from their perspective, including within the context of the COVID-19 pandemic. We identified these two participants to interview purposively as team members were in agreement they would be the most qualified to provide comprehensive insights on policy engagement given their senior leadership roles within MOLISA and extensive collaboration on our work. The intention of this descriptive case study is to capture factors related to this research and policy collaboration that interacted with external factors in the context of mental health policy development in Vietnam. We therefore did not seek to reach theoretical staturation but rather to capture the perspectives of core research and policy collaborators.

The semi-structured interview guide was developed by LC and JM with input from the study team with questions aligned with the three key recommendations for stakeholder engagement in GMH (Table 1) and other aspects (e.g., impact of COVID-19 pandemic on mental health policy priorities, whether results from the 2016-2019 SSM study helped inform the evaluation of the 1215 Program). Research team members (HM, NVC, LC) conducted the 60-min interviews with the assistance of an interpreter. Interviews were recorded with informed consent from participants and subsequently the interviews were transcribed and translated using forward-backward translation. Ethics approval for the activities conducted informing this case study was granted in Canada by Research Ethics BC, which oversees the harmonization of ethics application in BC (2018s0340) and in Vietnam by PHAD’s Institutional Ethics Review Board (2019/PHAD/IRIS-01).

### Data analysis

Team meeting minutes, focus group notes and translated interviews were analyzed using thematic analysis [29] with a code book that was informed by the stakeholder engagement recommendations [11]. Double-coding was conducted by co-first authors JM and LC, who completed coding and identified key themes arising from the data separately. Personal communications (emails and notes from conversations) with team members were similarly analyzed. All data coded separately by JM and LC from the different sources were then compared to ensure convergence of key themes through data triangulation [30]. Areas of divergence were discussed until agreement was made on the key themes. Key themes are detailed below.

## Results

Data from this case study demonstrate alignment with the three findings and recommendations for facilitating stakeholder engagement in GMH from the Murphy et al., framework [11]: (1) The importance of understanding context; (2) The nature of engagement, and (3) Communication and dissemination. Findings unique to this case study are described below, mapped onto the framework. The names and characteristics of the team members, focus group participants, and interview participants are anonymized for confidentiality purposes. The two interviewees are identified by number (1 and 2).

### The importance of understanding context

The case study confirms the importance of understanding the context within which researchers are working.

#### Invest in high quality formative research

A key finding from this case study was the importance of investing in high quality formative research from program inception to help the research team gain an in-depth contextual understanding of Vietnam. Focus group participants emphasized that the current policy and research partnership is an exemplary example of successes resulting from extensive initial collaborative work to develop a comprehensive understanding of the local cultural context. This includes understanding some of the barriers and drivers to mental health intervention implementation unique to Vietnam, such as factors influencing help-seeking, thus ensuring that SSM intervention can fit into the end-users’ setting. This work was led by researchers at UoM commencing in 1994, followed by the five research studies led by team members from SFU, PHAD, UoM, and UBC beginning in 2013 with the feasibility study that helped to examine and determine SSM’s appropriateness and acceptability in Vietnam.

Team meeting minutes and focus group participants emphasized that a key driver in the long history of collaboration was PHAD’s important role in helping the team to develop a comprehensive understanding of the local context. PHAD, as an non-governmental organization, has extensive social and political expertise, representing an intimate understanding of the implementation environment, including potential barriers and drivers for SSM uptake. PHAD also played an important role in helping to act as an interpreter for navigating through the local social and political environment and between various Vietnamese partners and collaborators, strengthening the team’s ability to implement the SSM intervention with cultural appropriateness.

#### Crises as a catalyst for positive change

Central to *the importance of understanding context* is the concept of leveraging barriers from challenging circumstances, in this case the COVID-19 pandemic, to create opportunities.

#### Increased recognition of mental health issues leading to increase in programs

There was an increase in and acknowledgement of mental health issues as a result of the COVID-19 pandemic. This highlighted the need for increased mental health services to address the severe shortage of support. As Interviewee 2 reported:

“The impacts of COVID-19: there is an increase in people who suffer from mental health problems such as students, women after giving birth. Therefore, there is a shortage of timely services to support families and women. The mental health care service system has not kept up with the impact of the pandemic both in health and social services.” (Interviewee 2)

Interviewee 2 also noted a troubling increase in severe mental illnesses, “After the COVID-19 pandemic, we noticed an increase in the number of people hospitalized for depression and mental disorders.”

Recognizing these challenges, the Government of Vietnam developed programs to increase mental health and other support to address the effects of the pandemic. The interview participants highlighted the important role of government to implement policies that provide programs and services not only to address mental health issues but also to assist with addressing the broader social determinants of health that impact mental health and general well-being. Programs also targeted specific vulnerable population groups, such as children and women. Interviewee 1 noted:

“The Vietnamese government also has many policies to support people in finding jobs or improving their lives to help people overcome the crisis. The government also has support policies for subjects such as children and women to overcome psychological crises. Our government also just signed a project to prevent mental disorders in children after the COVID-19 pandemic. We also have specific plans and policies in place to deal with the effects of this pandemic.” (Interviewee 1)

Furthermore, there was a focus on increasing human resource capacity to support population needs both in general and during the pandemic, and the critical role of government to achieve that goal. Capacity increases should also be focused at the local community level, as Interviewee 1 reported:

“We should ensure that there are enough community health staff, including [social] collaborators^1^, to be ready to solve community problems. The impact of COVID-19 is too fast and irregular. Medical and social systems didn’t adapt [in a] timely [way], lack of staff […] I think the role of government is extremely important in crisis response.” (Interviewee 1)

#### Crisis as catalyst for advancing digital mental health

An additional way in which the COVID-19 pandemic advanced mental health in Vietnam was through an increase in attention to the potential of digital technologies to expand access to mental health support. As Interviewee 2 reported, “Right after COVID-19 appeared, the Vietnamese government also thought that digital technology / online applications must be a priority” and that “in the upcoming time, we hope to have strong improvements in the application of digital technology in supporting mental health as well as providing psychological counseling for people suffering from stress, depression or symptoms of mental disorders.”

There was an appreciation for the potential of digital technologies to improve population mental health. Some initial changes during the pandemic included the implementation of “many online approaches such as working from home or consulting and taking care of people's health through the internet system” (Interviewee 2). Despite this, Interviewee 2 also noted that although “we have also implemented some content related to mental health, […] We find that currently in Vietnam, the application of digital technology is still very slow and there are not many digital applications in this field.” Interviewee 2 highlighted some of the challenges with digital approaches to health:

“However, because the context of COVID-19 was too fast, Vietnam did not have time to cope in the early stages. In addition, Vietnam's infrastructure is still very weak. It is very difficult for people to access health care information in the early stages. As we have gained more experience, the implementation of the telemedicine system has spread across the country. However, it also only covers areas with WiFi coverage and people [who] have access to smart technology such as phones and computers... For remote areas, this is still a challenge.” (Interviewee 2)

Interviewee 2 pointed further to the importance of inter-ministerial collaboration to support the delivery of digital mental health services:

“And now the government just stops at connecting telehealth between large hospitals and medical facilities at the district level. We have not been able to connect telehealth to people in communes. This issue is in the roadmap of the Ministry of Health in the coming time to implement telehealth, providing medical services to people.” (Interviewee 2)

Our research team aims to support the piloting and implementation of an Improved Mental Health Information System (IMHIS) in Vietnam in close collaboration with MOLISA. The goal is to help address gaps in MOLISA’s mental health information systems to enable them to effectively monitor and evaluate progress when implementing mental health innovations. Team meeting minutes captured the ongoing discussions with key MOLISA collaborators to secure project funding to help achieve this aim. Interviewee 2 highlighted the importance of “building systems such as data management as well as understanding the needs of service users to develop support policies for those objectives” to help address some of the challenges to widespread and unified electronic health information systems.

Our research team will implement and test the in-person SSM intervention to be delivered through a smartphone app (VMood) in late 2023. This shift to the digital format was driven by the Government of Vietnam’s prioritization of digital technologies for health as illustrated in the following quote from Interviewee 2:

“Currently, the Vietnamese government has a plan for digital transformation and has a document directing the enhancement of digital technology application to affiliated units. I think VMood is very much in line with the direction of the government. I think the implementation of Vmood application is very favorable in the near future.” (Interviewee 2)

### The nature of engagement

The Murphy et al., framework highlighted the importance of attention paid to processes impacting the nature of stakeholder engagement. We identified several key drivers related to this, as described below.

#### Invest adequate time and funding to support engagement and trust-building

iKT approaches to stakeholder engagement requires time and investment to support trust building and rapport. Focus group participants pointed to how the sustained and close collaboration with MOLISA resulted from nearly three decades of work that began with multiple mental health system development activities led by the UoM (from 1994 to 2013), continued program funding by GCC and CIHR culminating in the recently-funded studies to implement and scale-up VMood through to 2026, and a long term collaboration with implementing partner PHAD. Interviewee 1 indicated that”Yes, I think it's [cooperation between MOLISA and SFU and UoM] very effective. And after 2 years of hiatus from these activities due to the pandemic, I think more activities are needed.”

#### Create opportunities for meaningful engagement by policy makers

Focus group participants identified how the initial feasibility study (GCC: 2013-2016) helped act as a catalyst to establishing a partnership with the Government of Vietnam, specifically MOLISA and MoH, building on the momentum of MOLISA priorities to expand mental health care with the support of non-specialist providers such as social workers and social collaborators. The team has worked closely with MOLISA on subsequent funding proposals to continue ensuring project priorities are aligned with policy priorities. This sustained engagement of policy stakeholders throughout the whole research cycle, from inception to implementation, has helped to create real and meaningful policy change within Vietnam, culminating in the recent Prime Minister’s 1929 National Mental Health Program for period 2021-2030 that aims to strengthen community-based mental health services. As a result of these successes, the research-policy collaboration is, in turn, able to continue.

#### Mental health programs leading to improved inter-ministerial collaboration

Government policies have focused on increasing mental health programs, which require joint action by MoH and MOLISA to implement, enabling the two sectors to work more closely and effectively together and leading to improved inter-ministerial collaboration. As Focus Group participants mentioned, although joint efforts between the ministries have contributed to strengthening Vietnam’s mental health system, there are at times competing priorities and unique approaches for decision-making processes within the ministries. However, because mental health services in Vietnam are the responsibility of both sectors, this increased collaboration has been crucial to improved awareness of mental health and scope of services. As Interviewee 2 further reported:

“In localities, we have social protection centers for mental health care providers […]. In addition, the health sector also has hospitals that are responsible for treating patients with mental illnesses. On that basis, we also directed the implementation localities that there must be coordination and linkage in the treatment, care and nurturing of patients between the health facilities and social protection.” (Interviewee 2)

Development of the various mental health programs has also led to increased training and capacity development amongst medical and social workers to help expand the mental health workforce. For example, the Prime Minister’s 1929 Program has “allocated a budget line for training and capacity building for the contingent of social workers throughout the country. Therefore, we have a budget for this activity to train and improve the capacity of social work staff in all fields of digital technology” (Interviewee 2). Team meeting minutes have highlighted MOLISA’s commitment to capacity building through, among other targets and initiatives, training 60,000 social workers. Interviewee 2 describes some of the training programs in place to support capacity building:

“On a larger scale, there are cooperation programs in training fostering and improving the capacity of staff who work at social assistance facilities as well as medical staff who work in medical facilities. For example, at medical facilities, we have social work training programs for medical staff. As for social support facilities, we have training programs to support for mental health patients […]. Social and medical facilities are now cooperating very closely with each other in the care and treatment of people with severe mental health problems.” (Interviewee 2)

In alignment with the theme described above, the COVID-19 pandemic also acted as an incentive for improving inter-ministerial collaboration and has helped further highlight the need for improved inter-ministerial collaboration. This included regular meetings to develop and implement targeted programs prioritizing mental health, social work, and primary care. As Interviewee 1 reported:

“After 2 years of being hit by the pandemic, many urgent problems have arisen, we realized that it is very necessary to inter-sectorally coordinate between ministries. The government has a related strategy to deal with the pandemic, and the government’s coordination is also stronger on COVID-19 prevention. We [members of ministries] had great chances to meet each other on a regular basis. At present, we have more clear programs, for example the 1929 program on mental health, the 112 program on social work which have clearer regulations on goals, contents, solutions to achieve the goals, [and] we have a closer and closer relationship.” (Interviewee 1)

More specifically, Interviewee 1 spoke of increased collaboration between specific individuals within the two Ministries, leading to stronger relationships:

“The relationship between the two ministries has never been as good as it is at this time. COVID-19 pandemic helps to strengthen inter-sectoral coordination and especially in the field of primary care and MoH. In the previous period, apart from Deputy Minister [name], we had very few working relationships with other ministers and deputy ministers of the Ministry of Health. But now, we have a deeper comprehensive relationship. We can connect directly with deputy ministers such as Deputy Minister [name], Deputy Minister [name] and other deputy ministers of the health sector.” (Interviewee 1)

#### Leverage existing resources and relationships

Findings from this case study demonstrated the importance of leveraging existing resources to help advance MOLISA’s mandates in order to strengthen policy stakeholder engagement. The Government of Vietnam has clear mandates supporting their commitment to the Sustainable Development Goals by addressing the wider social determinants of health that impact mental health. For example, MOLISA is implementing a national target program on poverty reduction where they are “develop [ing] a series of policies such as […] vocational education so that people can have the opportunity to find jobs and escape poverty […] with invest[ments of] hundreds of billions of dong *[1 billion VND is equivalent to approximately 45,000 USD]* annually for poverty reduction” (Interviewee 2). This highlights how MOLISA’s mandate extends to initiatives that indirectly support and promote mental health and wellbeing. As a result, there have been changes to expert and public opinion on mental illness and treatment. Interviewee 1 spoke of some of these changes:

“Psychiatric facilities no longer confine people with mental health disorders, but are now switching to social institutions for rehabilitation care combining medicine and vocational training and job creation for mentally ill people […]. In the community, it is understood that the role of social work is very important to provide care for people with mental health problems. Additionally, combining psychology therapy in treatment, diagnosis, early detection [to] help effectively improve in mental health sector.” (Interviewee 1)

As described above, policy priorities have shifted to digital technologies for expanding mental health care to respond to challenges posed by the COVID-19 pandemic, including stress resulting from the lockdowns and social distancing measures. Despite the policy shift, Interviewee 1 acknowledged:

“However, at present, the training for social workers to use digital technology application to take care of people's health, we do not have any program to implement. If in the coming period, we are supported by the [VMood] project, we think this is a huge breakthrough in the field of mental health care for people and many beneficiaries of this service.” (Interviewee 1)

Focus group participants mentioned that while many of these policy changes were already taking place in Vietnam, our research collaboration helped act as a catalyst to advance MOLISA’s policy agenda on digital technologies and social worker training through securing external funding that continues to support policy initiatives. This applies both externally, with international research and funding partners and internally, with MOLISA and MoH. Our research-policy collaboration has been supported by committed policy makers who recognize the cooperative efforts required to address some of the challenges Vietnam faces. As Interviewee 2 reported,

“I think it’s a matter of perception of all levels of management: Realizing the importance of coordination and mutual support. In health care and support for people with mental illness, I see great attention from leaders of the two ministries. Leaders of the two ministries are very determined to direct in this field of cooperation to bring about the best results in the treatment, care and nurturing of mental [health] patients, minimizing problems of depression and mental stress for them.” (Interviewee 2)

Interviewee 1 stated, “we greatly appreciate the cooperation with the University of Melbourne and Simon Fraser University,” emphasizing the degree of trust and rapport that has been built. This was also highlighted by the focus group participants and team meeting minutes. In addition, meeting minutes emphasized the critical element of committed policy makers in ensuring project and collaboration success, without which our research team would have been severely restricted in intervention implementation.

Crucially, focus group participants also mentioned how an invaluable partnership with PHAD, our implementing partner, has been key to the success of this ongoing work. The Co-Principal Investigator of this research program (NVC) is the Deputy Director at PHAD and he has been instrumental in helping the team navigate the political landscape in Vietnam throughout the long history of the research programs. NVC has been a key interpreter to helping all parties avoid misunderstandings.

#### Commitment to partnerships and sustained engagement

An additional driver was the commitment by all partners to long-term collaboration and engagement. This was evident in the team meeting minutes and focus group discussions. As one participant indicated, the partnership with MOLISA has been a genuine engagement spanning a decade. MOLISA has provided matched funding for two of the GCC-funded Transition to Scale projects, and has been instrumental with providing project support, most recently with commitment to social worker training to deliver remote coaching for the digital VMood intervention. Focus group participants spoke of how this dedication was facilitated in part by the research team engaging with MOLISA throughout the research process, promoting their empowerment and active participation.

Focus group participants also emphasized how the commitment to partnerships by our funders AP, GCC, and CIHR has been instrumental to providing the required support for sustained program development and implementation. Program managers within the agencies have been extremely supportive and encouraging of our work. Without their ongoing and instrumental support this collaborative work would not have been possible.

#### Reciprocity

Lastly, reciprocity is a key driver related to the nature of engagement. Our international and interdisciplinary research team had substantial capacity for developing valuable, timely, and competitive research proposals. The early phase was funded by AusAID and AP, followed by GCC, with current funding from both CIHR and GCC (through to 2026). Through multiple rounds of successfully securing ongoing funding from the various funding programs, the research team was able to contribute funding to support the development and implementation of MOLISA’s programs. This includes using findings from our GCC-funded SSM study to help inform an evaluation of the 1215 National Program (2011-2020) proposal for mental health and development of policy priorities for the subsequent 1929 (2021-2030) proposal, which MOLISA was required to submit to secure continued funding from the central government. Interviewee 2 provided further examples of how the collaborative partnership has been helpful to advancing the Government of Vietnam’s mandates on mental health capacity development:

“The bi-direction of cooperation [and] problem solving. For example, there [is a] lack of technical skills in mental health in Vietnam. Professors and two universities have supported Vietnam. The mechanism of cooperation is very responsive and timely. The training programs of professors [name] and [name] are very suitable. Study visits [examining] Australian law [and] Canada’s mental health program are very effective. Mobiliz[ing] financial resources from Canada, [these] important fund[ing] sources help a lot to solve technical problems, design and provide technical advice and organize a direct exchange. [These are] strengths and important lessons.” (Interviewee 2)

Importantly, focus group participants and team meeting minutes highlighted how the research team would not have received the continuous funding without MOLISA’s commitment and critical contributions on the grant proposals, along with facilitating intervention implementation and scale-up. Interviewee 1 similarly acknowledged how the reciprocal nature of the relationship helped to maintain support from the Vietnamese Government:

“I think there must be a commitment between the two sides, especially from the Vietnamese government. The Department of Social Protection is also ready to actively support this cooperation activity because when the project is implemented, it will bring a lot of benefits and efficiency to Vietnamese people, especially mental health issues. For MOLISA, we have a unified direction from top to bottom through all levels. I believe these activities are implemented effectively.” (Interviewee 1)

### Communication and dissemination

Communication and dissemination are important factors to promoting uptake of mental health interventions for successful implementation and sustained policy stakeholder engagement. Strategies supporting these efforts are described below.

#### Invest in informed mental health awareness raising and communication strategies

Although Vietnam has made substantial progress in mental health policy development for initiatives to strengthen population mental health, there remains limited awareness in the community. This hinders understanding of mental health treatment and help-seeking. Mental health awareness-raising campaigns are also important to promote understanding and support at home. Interviewee 1 spoke of the importance of this:

“I think in mental health we should develop apps for people to do online. But it is important for family members to understand, register and support the patients because of the patient’s awareness. The problem is that our project should include this part to raise awareness for the project areas, assign tasks to medical facilities to develop this app. In this app there is a section about health and a section about social worker to work together.” (Interviewee 1)

Research team members have contributed in various ways to help increase mental health awareness in the community. For example, UoM partners have developed various training and capacity building programs and research leads have presented at numerous national MOLISA and MoH meetings, with attendance from all 63 provinces and municipalities. The Government of Vietnam has facilitated these awareness-building initiatives and provided support to advance mental health reform within the country. As a result of investing in awareness raising and communication strategies, there has been a substantial change in mental health policy, care, and attitudes from one of institutionalization with the belief that individuals with mental illness need to be institutionalized, as described in the quotation above. Further, there is an understanding that often mental illnesses can be addressed successfully in the community. Interviewee 1 reflected on this:

“In addition, Vietnamese people now no longer think that people with mental health problems are [only people with] schizophrenia as in the past. They understand that people with mental health problems may be people with depression, behavioral disorders. In the community, it is understood that the role of social work is very important to provide care for people with mental health problems. Additionally, combining psychology therapy in treatment, diagnosis, early detection [to] help effectively improve [the] mental health sector.” (Interviewee 1)

#### Create mechanisms to support engagement from program inception

Related to the *nature of engagement – reciprocity* finding, the team has utilized an iKT approach, creating mechanisms to provide opportunities for policy stakeholder engagement in research from the beginning. This includes involving policy leaders in funding proposal development to ensure project aims are aligned with policy priorities. Focus group participants spoke of this, emphasizing how MOLISA collaborators have been heavily engaged in grant proposal development since the GCC SSM proposal. The research team has also engaged policy stakeholders in knowledge dissemination activities, such as journal publications and conference presentations to ensure findings relevant for their country are widely and appropriately disseminated.

#### Invest in capacity development opportunities to support knowledge translation and communication activities by researchers

Throughout the duration of the research program, there has been meaningful inclusion of trainees at all stages of their educational programs, from undergraduate students to post-doctoral fellows. Trainees have been provided with ongoing mentorship support from the interdisciplinary research team and have in turn led peer-reviewed journal articles, presented at conferences, and contributed to funding proposal development. Given the unique nature of the research-policy collaboration, trainees have also been provided with unique opportunities for real capacity building in global policy stakeholder engagement, with regular opportunities to interact with MOLISA and other partners.

#### Invest in activities that promote mental health capacity building of policy makers

Lastly, dedicated iKT processes have supported activities promoting capacity building of policy makers within Vietnam, leading to among other things, an increase in knowledge and skills. Meeting minutes and focus group participants highlighted how this process began prior to our research projects with the initiatives led by the UoM partners, including work with WHO and mental health leadership development. Interviewee 1 summarized:

“Historically, in 2009-2010, when Professor [name] worked with WHO to support Vietnam in summarizing the assessment of the mental health care sector of the labor invalids and social sector, Professor's suggestions have been followed up in project 1215 and are very important technical advices to build [social worker capacity]. The social sector is well built in terms of theoretical framework as well as the program to support for people with mental health [conditions]. In that context, there is a lack of a legal framework and a program framework [so] Professor [name] and WHO’s comments for the period 2012-2020 are very important and project 1215 achieved a lot of success. Thanks to the technical support of Prof. [name], the situation in Vietnam has improved a lot in the sector of mental health […]. In the past period, study tours and training courses of SFU and Melbourne University have trained senior researchers [and have been] effective in raising awareness, knowledge and skills for key local officials.” (Interviewee 1)

The long history of the research-policy collaboration has culminated in the recent 1929 program, which was developed “based on the success of the 1215 program (Interviewee 1)” and supported by technical and practical suggestions from our research team. The 1929 program represents substantial changes to Vietnamese mental health policy and highlights the continued dedication from the Vietnamese Government to improving community-based mental health services to support their country’s mental health.

## Discussion

This case study describes a decade-long research and policy collaboration between researchers in Canada, Australia and Vietnam and MOLISA policy makers that continues to advance community-based mental health policy and practice in Vietnam. The results identify factors from within the collaboration that have contributed to mental health policy development in Vietnam, and external mediating factors that have both advanced and challenged mental health policy progress. Though policy engagement is recognized as central to facilitating the implementation of evidence-based policy and practice [31], there is limited literature describing best practices for promoting sustained policy engagement in GMH [32]. We have drawn on Murphy et al.’s [11] facilitators of stakeholder engagement in GMH as the analysis framework. Though the results describe findings from Vietnam and the context of this specific collaboration, they also offer lessons learned that may help to support successful research and policy collaborations to advance mental health policy development in other contexts.

### The importance of understanding context

Investing in the project inception phase, which may include collaborative funding proposal development, initial pilot studies, and formative research such as situational and stakeholder analyses, is one important element of understanding context and should not to be rushed or overlooked [33]. Formative research can help to identify appropriate stakeholders [34], including policy champions, and allows time to ensure alignment with local priorities. The formative phase not only helps to establish an understanding of factors like the feasibility and acceptability of interventions, but also allows time to build and strengthen trusting and reciprocal relationships, facilitating the involvement of policy partners. Engaging other key partners including people with lived experience, community members, and health care providers, can help to further promote appropriateness, responsiveness and sustainability of policies and programs [11, 35].

Despite the many challenges related to the COVID-19 pandemic, the results demonstrate that it has also acted as a catalyst to advance the prioritization of mental health by the government of Vietnam. The role of crises as catalysts for mental health system development has been recognized in many contexts prior to the pandemic. For example, the concept of ‘building back better’ has been widely applied in relation to supporting mental health systems in the wake of emergency situations [36]. The scope of the pandemic and its impact on health systems and mental health needs globally represents an unprecedented indicator of the universal and urgent need to strengthen mental health systems [37]. In Vietnam, the pandemic has increased political awareness of the urgency of expanding mental health care and has in turn sparked momentum for mental health system strengthening through an increase in inter-ministerial collaboration which previously often acted as a barrier to mental health system development in the country [18].

In addition to increasing direct mental health support, the pandemic has also galvanized action by MOLISA towards addressing the social determinants of health. This indicates the importance of engaging and collaborating with policy stakeholders beyond the health sector, with MOLISA’s mandate for social protection contributing to their ability to take this broader approach. The importance of multisectoral engagement is recognized as an important step to promoting a ‘health in all policies’ approach to public health [38] and as essential to strengthening mental health systems [37]. Additional engagement, including with education, economic development and other related sectors, could therefore advance a population-health approach to supporting mental health protection and promotion and further enhance community-based care in Vietnam.

Finally, an increased reliance on digital technologies, including to support mental health, developed globally during the pandemic [39, 40]. Our team shifted towards the adaptation of an in-person depression intervention for app-based delivery as a response to the government’s interest in strengthening digital health even prior to the pandemic. The pandemic has accelerated the urgency of enhancing digital mental health. The ‘digital divide’ [41] resulting from lack of access to Internet and digital devices among underserved populations is a global challenge. As access to services, including mental health support, shifts more towards digital technologies, it will be imperative that the government of Vietnam invests in infrastructure to promote access, particularly among the most vulnerable. It will also remain essential to invest in accessible and evidence-based face-to-face mental health supports, as digital options are not always preferred or appropriate [42].

### Nature of engagement

An investment in the quality of partnerships from the beginning is essential to a strong and longstanding collaboration. This requires both dedicated time and funding to support engagement and trust building, enabling partners to work towards common goals. Throughout the decade of work in Vietnam, our research team has been committed to utilizing iKT approaches to policy stakeholder engagement, initiating collaborative activities with shared decision-making reflecting diverse perspectives from program inception, which has been shown to support the development of a common vision focusing on mutual goals [43]. This has helped to ensure program relevance and commitment to scale-up by identifying synergies with other policy priorities, recognized as a key factor in stakeholder engagement [44]and supporting the duration and quality of the research-policy collaboration. Similar to findings from Murphy et al. [11], results demonstrated that iKT approaches take substantial time and investment from all partners, but are integral to promoting trust building, rapport, and reciprocity, ensuring policy partners are empowered and remain engaged.

A common barrier to stakeholder engagement is limited resources [45]. Findings from this case study supported this and indicated that another factor of sustained policy engagement is the funding landscape. Global mental health research funding cycles are often brief, limiting the ability of researchers to build long-term, trusting relationships with partners [11]. The continuous funding for this research program, supported in large part by GCC’s funding model which includes opportunities to apply for funding across a trajectory ranging from formative research to ‘transition-to-scale’ and beyond, has been essential. Similarly, CIHR recognized the importance of using digital methods to expand access to an evidence-based mental health intervention by providing subsequent funding to develop and test VMood. Our engagement with MOLISA has been equally important in helping to secure sustained funding as evidence of meaningful policy engagement and government commitment, including via providing matched funds for two of the GCC-funded Transition to Scale projects. The multiple rounds of funding have helped to further existing government priorities in Vietnam, including for developing mental health programs that require joint action by MoH and MOLISA. Inter-ministerial collaboration has historically been challenging, resulting in a lack of coordinated mental health and social care systems [18]. However, the two Ministries have worked together increasingly over the past decade on shared initiatives supporting population mental health.

Other key drivers related to the nature of engagement include a genuine commitment to long-term partnerships and meaningful engagement from all partners, including funding agencies, which enabled everyone to work towards common goals. The importance of engagement of policy stakeholders in the entire research process, which requires commitment and dedicated resources, has been emphasized in the literature as essential for GMH implementation [44, 46–48]. Sustained “engaged participation” has been described as the highest level of stakeholder engagement [48]. The research team has actively engaged with MOLISA throughout the research process, facilitating a truly collaborative process. Leveraging existing resources and relationships is also a key driver. The relationship with local research and implementation partner PHAD was essential to the success of this collaboration. PHAD has a longstanding relationship with government partners and has been active in population and public health research across many priority areas for over a decade. Collaboration with local experts who have established relationships with key policy stakeholders and fully understand the nuances of the social and political landscape is fundamental to navigating and facilitating policy collaboration [11].

### Communication and dissemination

Several approaches to communication with core partners and dissemination of research evidence act as drivers of mental health policy development. First, informed mental health awareness-raising and communications strategies are a recommended approach for supporting stakeholder engagement in mental health policies and interventions [11]. Beyond direct policy impact, MOLISA partners identified the potential for the VMood project to increase mental health awareness among the general population through engagement with community members via app use and with social workers who will be providing supportive coaching for VMood users. Awareness about common mental disorders and mental health help-seeking is very low in Vietnam [19]. Effective communication and awareness raising strategies have been shown to increase help-seeking behaviours and to have other positive outcomes including decreasing stigma [49].

Capacity building via several knowledge dissemination approaches has been fundamental to policy engagement and impact in Vietnam. Our partnership has taken an iKT approach, whereby ‘knowledge users’ with the ability to implement change are involved in every stage of the research process [13]. A key emphasis of iKT is capacity building for knowledge users “in the most effective creation of knowledge and its translation into action” [50]. In the case of this research-policy collaboration, the engagement of MOLISA partners in research publications and in other capacity building initiatives including mental health leadership workshops led by UoM [51] has had a direct impact on mental health policy. Results of a systematic review about policy engagement interventions in GMH suggest that such leadership programs led to increased engagement by participants in mental health system strengthening in their jurisdictions [32]. MOLISA, MoH and the Ministry of Finance have also participated in study tours, hosted by study team members, to Australia and Canada to learn about aspects of their health and social services sectors. As described by MOLISA respondents, their participation in capacity building initiatives and long-term collaboration with the study team led to a shift in perceptions about mental illness requiring institutionalization to an understanding of the importance of community-based approaches to care. Tangible progress is evident with the 1929 program which was signed by the Prime Minister in late 2020, renewing a commitment to mental health system strengthening in Vietnam.

## Limitations

This paper presents a descriptive case study that identifies drivers and barriers related to policy engagement in GMH to support the enhancement of community-based mental health policy and practice in Vietnam. For this case study we have drawn on the perspectives of study team members and two senior policy partners in MOLISA as we focused on policy makers who were closely involved in this work. We therefore did not capture perspectives from the broader policy environment and did not seek to reach theotectical saturation. We have not employed systematic measures of policy engagement success or policy change. We believe, however, that reflecting on the long history of engagement in Vietnam and drawing on the depth of expertise of all parties has allowed us to accurately describe the key factors both within the collaboration and in the external environment that have contributed to ongoing policy engagement and related changes in care for common mental health conditions in Vietnam.

## Conclusions

The findings from this case study identified a number of key drivers and barriers to policy engagement in GMH in an LMIC setting. Engagement with policy leaders is crucial to ensuring alignment of research goals with local priorities, in turn supporting uptake and scale-up of innovations. Findings highlight the importance of sustained and meaningful policy stakeholder engagement from the formative stages of GMH research and throughout program implementation. This was facilitated by leveraging existing relationships and resources, but requires commitment and active participation from all partners to ensure the collaboration is built on trust and addresses key policy priorities identified by local stakeholders.

The findings also point to the integral role that funding agencies play in helping to ensure sufficient and long-term resources for research-policy collaborations to develop rapport and enhance trust-building and capacity development, allowing all partners to contribute in a meaningful way to advance a shared goal. Continuous funding enables partnerships to thrive, and in turn supports effective development, testing, and implementation of evidence-based mental health interventions. As mental health is a leading contributor to the global burden of disease, it is imperative that GMH research continues engaging various stakeholders, including policy partners, to ensure implementation and scale-up of mental health innovations to promote and sustain population mental health. Future research could evaluate the degree of policy stakeholder engagement and its association with implementation outcomes, thus helping to not only identity, but also quantify best practices for policy stakeholder engagement in GMH research.

### Supplementary Information

**Additional file 1.** Interview Questions.

## Data Availability

The datasets used and/or analysed during the current study are available from the corresponding author on reasonable request.

## Abbreviations

HICs: High Income Countries
WHO: World Health Organization
LMICs: Low-and-Middle Income Countries
iKT: Integrated Knowledge Translation
GMH: Global Mental Health
SFU: Simon Fraser University
UBC: University of British Columbia
UoM: University of Melbourne
PHAD: Institute of Population, Health and Development
MOLISA: Ministry of Labour, Invalids and Social Affairs
MOH: Ministry of Health
CMHP: Community Mental Health Program
AusAID: Australian Agency for International Development
AP: Atlantic Philanthropies
GCC: Grand Challenges Canada
SSM: Supported Self-Management
RCT: Randomized Controlled Trial
CIHR: Canadian Institutes of Health Research

## Footnotes

1 Social collaborators are a group of lay social workers unique to Vietnam, mobilized in the community to provide support to community members. They are primarily the responsibility of MOLISA.
