# Supplementary material for: An integrated knowledge translation (iKT) approach to advancing community-based depression care in Vietnam: lessons from an ongoing research-policy collaboration
Source: BMC Health Serv Res. 2024 Jan 27;24:142. doi: 10.1186/s12913-023-10518-3 (PMC10821570; doi:10.1186/s12913-023-10518-3)
Supplement: Supplementary file 1 — Additional file 1. Interview Questions. [file 12913_2023_10518_MOESM1_ESM.pdf]

Dear [name]

RE: Vietnam Mental Health Projects (MAC-FI / IRIS-DSV) - Interview Questions

We are writing this letter to invite you to participate in an interview with members of the Vietnam Mental Health Projects Team. We would like to learn more about the long history of policy engagement in Vietnam that has led to sustained promotion of mental health at a population level, including a commitment to community-based approaches. Appended below are the proposed interview questions. We anticipate this interview to take approximately one hour.

Interview Questions:

1. Could you please describe your role and responsibilities within MOLISA?
2. Could you please describe the Vietnamese Government's commitment to the United Nations Sustainable Development Goals - specifically for SDG1 (End Poverty), SDG 8 (Decent Work and Economic Growth) and SDG10 (Reduced Inequalities)?
    a. What policies and programs has MOLISA developed and implemented to meet the Goals?
3. We are interested in capturing the key ingredients that have enabled and supported a sustained collaboration between the Government of Vietnam/ MOLISA and our team over the last decades. Based on your experience:
    a. Could you please provide a brief history of this relationship?
    b. What has your role been?
    c. From the point of view of MOLISA, what factors have enabled and supported this long-term collaboration?
    d. Are these factors the same or different to those that have supported collaboration between MOLISA and other international or local organisations?
    e. What are the key factors that have enabled this collaboration to be sustained in the long term?
    f. What challenges have there been in building and sustaining collaborations, particularly with international organisations?
4. Did the results from the MAC-FI study help inform the evaluation of the 1215 2011-2020 program?
    a. If yes, how?
5. Did the results from the MAC-FI study inform the development of the 1929 2021-2030 proposal, including the policy priorities that were included in the proposal (e.g., commitment to community-based mental health services)?
    a. If yes, how?
6. Did MOLISA's broader collaboration with SFU/UOM/PHAD (beyond the MAC-FI study) inform the development of the 2021-2030 proposal, including the policy priorities that were included in the proposal (e.g., commitment to community-based mental health services)?

a. If yes, how?

7. Could you please describe the government commitment to mobile health technology for mental health service delivery, and the challenges that are foreseen in implementation of digital approaches to mental health service delivery?
8. More specifically, is there a government commitment to implement our mobile application VMood that we adapted from the in-person supported self-management intervention we tested in the MAC-FI study?
9. Could you please describe the current extent and nature of cooperation between MOLISA and MoH in development and implementation of community-based mental health policy and practice in Vietnam?
10. Has the extent of nature of cooperation between MOLISA and MoH changed during the last decade? If yes:
    a. In what ways has the extent and nature of cooperation changed?
    b. What factors have contributed to the change in the extent and nature of cooperation?
    c. Has our collaborative work influenced the extent and nature of cooperation between MOLISA and MOH? If yes, in what way(s)?
11. Could you please describe how the COVID-19 pandemic has impacted policy priorities related to mental health? How has the pandemic impacted our work together? How has it influenced mHealth development?
12. Based on our collaborative work to improve community-based mental health services in Vietnam, what would you say are the ingredients to a successful research/ policy collaboration? How could this be helpful to other similar collaborations?

Thank you for your consideration.
